# Poly[[μ-aqua-diaqua­bis­(μ-furan-2,5-dicarboxyl­ato-κ^2^ *O* ^2^:*O* ^5^)bis­(1,10-phenanthroline-κ^2^ *N*,*N*′)dicopper(II)] *N*,*N*-dimethyl­formamide monosolvate]

**DOI:** 10.1107/S1600536812046041

**Published:** 2012-11-14

**Authors:** Ya-Feng Li, Xiao-Lin Qin, Yue Xu, Yong-Peng Yuan, Wen-Yuan Gao

**Affiliations:** a School of Chemical Engineering, Changchun University of Technology, Changchun 130012, People’s Republic of China

## Abstract

The asymmetric unit of the title compound, {[Cu_2_(C_6_H_2_O_5_)_2_(C_12_H_8_N_2_)_2_(H_2_O)_3_]·C_3_H_7_NO}_*n*_, contains two Cu^II^ atoms, two furan-2,5-dicarboxyl­ate (*L*) ligands, two 1,10-phenanthroline (phen) ligands, three coordinating water mol­ecules and one *N*,*N*-dimethyl­formamide solvent mol­ecule. Each Cu^II^ atom is coordinated by two N atoms from one phen ligand, two O atoms from two *L* ligands and two water mol­ecules in a distorted octa­hedral geometry. The main difference between the environments of the two independent Cu atoms is in the Cu—O_water_ distances, which are 2.415 (2) and 2.639 (2) Å for one Cu^II^ atom and 2.3560 (19) and 2.911 (4) Å for the other. Ligands *L* and one independent water mol­ecule bridge the Cu^II^ atoms, forming corrugated polymeric layers parallel to the *ab* plane. Inter­molecular O—H⋯O and C—H⋯O hydrogen bonds consolidate the crystal packing.

## Related literature

For a related structure, see: Li *et al.* (2012 ▶).
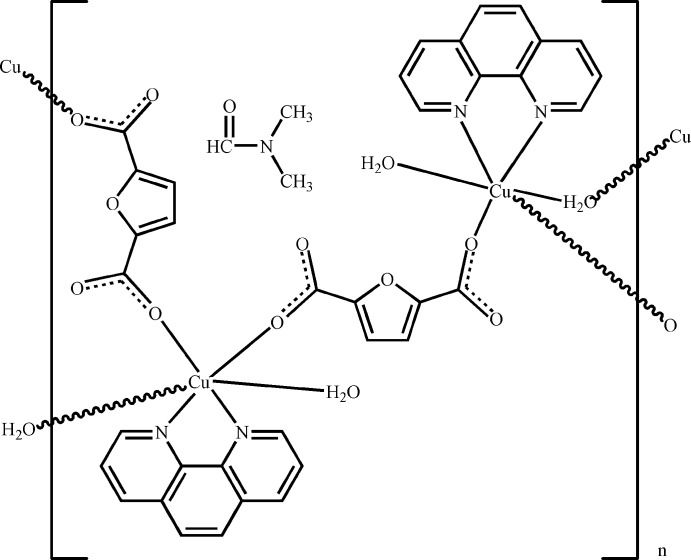


## Experimental

### 

#### Crystal data

[Cu_2_(C_6_H_2_O_5_)_2_(C_12_H_8_N_2_)_2_(H_2_O)_3_]·C_3_H_7_NO
*M* *_r_* = 922.78
Monoclinic, 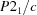

*a* = 16.620 (3) Å
*b* = 11.600 (2) Å
*c* = 20.168 (4) Å
β = 92.41 (3)°
*V* = 3884.6 (13) Å^3^
*Z* = 4
Mo *K*α radiation
μ = 1.17 mm^−1^
*T* = 293 K
0.35 × 0.20 × 0.17 mm

#### Data collection

Rigaku R-AXIS RAPID diffractometer
Absorption correction: multi-scan (*ABSCOR*; Higashi, 1995 ▶) *T*_min_ = 0.685, *T*_max_ = 0.826
36308 measured reflections
8855 independent reflections
6642 reflections with *I* > 2σ(*I*)
*R*_int_ = 0.047

#### Refinement

*R*[*F* ^2^ > 2σ(*F* ^2^)] = 0.041
*wR*(*F* ^2^) = 0.110
*S* = 1.05
8855 reflections
561 parameters
13 restraints
H atoms treated by a mixture of independent and constrained refinement
Δρ_max_ = 0.65 e Å^−3^
Δρ_min_ = −0.35 e Å^−3^

### 

Data collection: *PROCESS-AUTO* (Rigaku, 1998 ▶); cell refinement: *PROCESS-AUTO*; data reduction: *CrystalStructure* (Rigaku/MSC, 2002 ▶); program(s) used to solve structure: *SHELXS97* (Sheldrick, 2008 ▶); program(s) used to refine structure: *SHELXL97* (Sheldrick, 2008 ▶); molecular graphics: *DIAMOND* (Brandenburg, 2000 ▶); software used to prepare material for publication: *SHELXL97*.

## Supplementary Material

Crystal structure: contains datablock(s) I, global. DOI: 10.1107/S1600536812046041/cv5353sup1.cif

Structure factors: contains datablock(s) I. DOI: 10.1107/S1600536812046041/cv5353Isup2.hkl

Additional supplementary materials:  crystallographic information; 3D view; checkCIF report

## Figures and Tables

**Table 1:** Hydrogen-bond geometry (Å, °)

*D*—H⋯*A*	*D*—H	H⋯*A*	*D*⋯*A*	*D*—H⋯*A*
O1*W*—H1*A*⋯O5^i^	0.84 (2)	1.88 (2)	2.715 (3)	170 (3)
O1*W*—H1*B*⋯O7	0.85 (2)	1.96 (2)	2.729 (3)	151 (3)
O2*W*—H2*A*⋯O10^ii^	0.88 (2)	1.98 (2)	2.729 (3)	143 (2)
O2*W*—H2*B*⋯O3*W* ^i^	0.86 (2)	1.89 (2)	2.726 (3)	163 (3)
O3*W*—H3*A*⋯O2	0.85 (2)	2.14 (3)	2.694 (3)	123 (2)
O3*W*—H3*B*⋯O41^iii^	0.88 (2)	1.88 (2)	2.758 (4)	178 (4)
C3—H3⋯O5^i^	0.93	2.53	3.424 (3)	161
C4—H4⋯O2^i^	0.93	2.44	3.287 (3)	152
C9—H9⋯O10^iv^	0.93	2.33	3.193 (3)	155
C10—H10⋯O7^iv^	0.93	2.41	3.324 (3)	168
C15—H15⋯O8^v^	0.93	2.42	3.327 (4)	166
C18—H18⋯O5^iii^	0.93	2.56	3.416 (4)	154
C20—H20⋯O2^iii^	0.93	2.52	3.230 (4)	133
C21—H21⋯O41	0.93	2.54	3.349 (5)	146
C33—H33⋯O2*W* ^vi^	0.93	2.59	3.402 (5)	146

Symmetry codes: (i) 
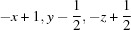
; (ii) 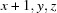; (iii) 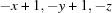; (iv) 
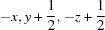
; (v) 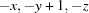; (vi) 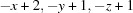.

**Fig. 1.**
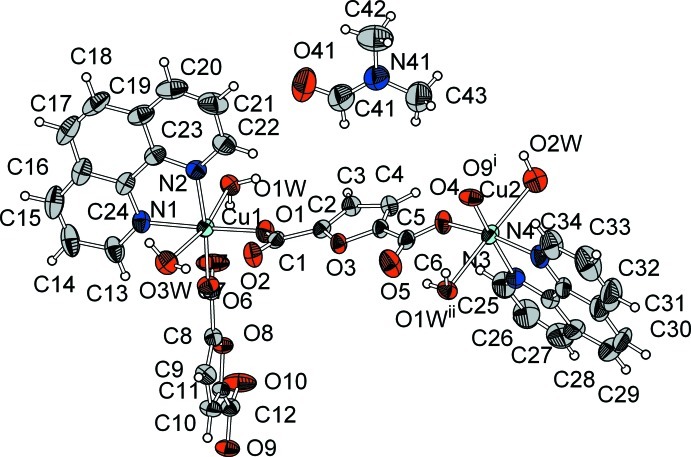
View of (I), showing the atomic labelling scheme and displacement ellipsoids drawn at the 50% probability level [symmetry codes: (i) 1 + x, y, z; (ii) 1 - x, 0.5 + y, 0.5 - z.]

**Fig. 2.**
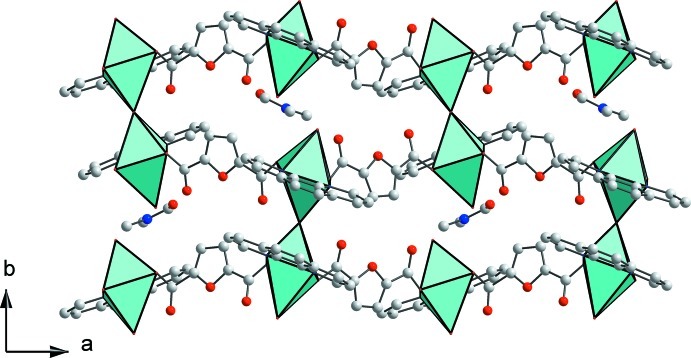
A portion of the crystal packing of (I) viewed along the c axis and showing the coordination environment of Cu centers as polyhedron.

## supplementary crystallographic information

### Comment

As the analogous structure of BDC (benzene-1,4-dicarboxyl acid), FDA (furan-2,5-dicarboxyl acid) attracts attention owing to the bond angle of two carboxyl groups about 126°. Recently, we utilized furan-2,5-dicarboxyl acid as the ligand to construct coordination polymers (Li *et al.*, 2012). As an extension of that work, herewith we present the crystal structure of the title compound, [[Cu*L*(H_2_O)(phen)]_2_^.^H_2_O]_n_^.^nDMFA (*L* = furan-2,5-dicarboxylato, phen = 1,10-phenanthroline, DMFA = *N*,*N*-dimethylformamide) (I).

The asymmetric unit of (I) contains two Cu^II^ cations, two anionic ligands *L*, two ligands phen, three coordinated water molecules and one DMFA solvent molecule (Fig.1). Each copper center is coordinated by two N atoms from one phen ligand, two O atoms from two ligands *L* and two water molecules in a distorted octahedral geometry. The main difference in environment of two independent Cu atoms is the difference in Cu—O_water_ distances, which are equal to 2.415 (2) and 2.639 (2) Å for one Cu atom and 2.3560 (19) and 2.911 (4) Å for another. Ligands *L* and one independent water molecule bridge copper centers into corrugated polymeric layers prallel to *ab* plane (Fig.2). Intermolecular O—H···O and C—H···O hydrogen bonds (Table 1) consolidate the crystal packing.

### Experimental

Furan-2,5-dicarboxyl acid (0.0156 g, 0.10 mmol), Cu(NO_3_)_2_^.^3H_2_O (0.0244 g, 0.10 mmol), and 1,10-phenanthroline (0.0180, 0.10 mmol) were dissolved in DMFA (5 ml, 75 mmol) under stirring. The mixture with molar ratio of 1 (furan-2,5-dicarboxyl acid): 1 (Cu(NO_3_)_2_^.^3H_2_O): 1 (1,10-phenanthroline): 750 DMFA was layed under room temperature for 8 days. The blue block product was collected as a single phase.

### Refinement

Water H atoms were located in a difference Fourier map and refined with restraints O—H = 0.86 (2) Å, and *U*_iso_(H) = 1.2*U*_eq_(O). C-bound H-atoms were placed in calculated positions (C—H 0.93–0.96 Å), and were included in the refinement in the riding-model approximation, with *U*_iso_(H) = 1.2Ueq(C).

### Crystal data

**Table:**

[Cu_2_(C_6_H_2_O_5_)_2_(C_12_H_8_N_2_)_2_(H_2_O)_3_]·C_3_H_7_NO	*F*(000) = 1888
*M**_r_* = 922.78	*D*_x_ = 1.578 Mg m^−^^3^
Monoclinic, *P*2_1_/*c*	Mo *K*α radiation, λ = 0.71073 Å
Hall symbol: -P 2ybc	Cell parameters from 2000 reflections
*a* = 16.620 (3) Å	θ = 1.2–27.5°
*b* = 11.600 (2) Å	µ = 1.17 mm^−^^1^
*c* = 20.168 (4) Å	*T* = 293 K
β = 92.41 (3)°	Block, blue
*V* = 3884.6 (13) Å^3^	0.35 × 0.20 × 0.17 mm
*Z* = 4	

### Data collection

**Table:**

Rigaku R-AXIS RAPID diffractometer	8855 independent reflections
Radiation source: fine-focus sealed tube	6642 reflections with *I* > 2σ(*I*)
Graphite monochromator	*R*_int_ = 0.047
Detector resolution: 10.00 pixels mm^-1^	θ_max_ = 27.5°, θ_min_ = 1.2°
ω scans	*h* = −20→21
Absorption correction: multi-scan (*ABSCOR*; Higashi, 1995)	*k* = −15→15
*T*_min_ = 0.685, *T*_max_ = 0.826	*l* = −26→26
36308 measured reflections	

### Refinement

**Table:**

Refinement on *F*^2^	Primary atom site location: structure-invariant direct methods
Least-squares matrix: full	Secondary atom site location: difference Fourier map
*R*[*F*^2^ > 2σ(*F*^2^)] = 0.041	Hydrogen site location: inferred from neighbouring sites
*wR*(*F*^2^) = 0.110	H atoms treated by a mixture of independent and constrained refinement
*S* = 1.05	*w* = 1/[σ^2^(*F*_o_^2^) + (0.0551*P*)^2^ + 1.1821*P*] where *P* = (*F*_o_^2^ + 2*F*_c_^2^)/3
8855 reflections	(Δ/σ)_max_ = 0.001
561 parameters	Δρ_max_ = 0.65 e Å^−^^3^
13 restraints	Δρ_min_ = −0.35 e Å^−^^3^

### Special details

**Table:**

Geometry. All e.s.d.'s (except the e.s.d. in the dihedral angle between two l.s. planes) are estimated using the full covariance matrix. The cell e.s.d.'s are taken into account individually in the estimation of e.s.d.'s in distances, angles and torsion angles; correlations between e.s.d.'s in cell parameters are only used when they are defined by crystal symmetry. An approximate (isotropic) treatment of cell e.s.d.'s is used for estimating e.s.d.'s involving l.s. planes.
Refinement. Refinement of *F*^2^ against ALL reflections. The weighted *R*-factor *wR* and goodness of fit *S* are based on *F*^2^, conventional *R*-factors *R* are based on *F*, with *F* set to zero for negative *F*^2^. The threshold expression of *F*^2^ > σ(*F*^2^) is used only for calculating *R*-factors(gt) *etc*. and is not relevant to the choice of reflections for refinement. *R*-factors based on *F*^2^ are statistically about twice as large as those based on *F*, and *R*- factors based on ALL data will be even larger.

### Fractional atomic coordinates and isotropic or equivalent isotropic displacement parameters (Å^2^)

**Table:**

	*x*	*y*	*z*	*U*_iso_*/*U*_eq_	
Cu1	0.262045 (16)	0.46824 (3)	0.095417 (14)	0.03123 (9)	
Cu2	0.778317 (16)	0.54556 (3)	0.415298 (14)	0.03424 (10)	
O1	0.34174 (10)	0.45626 (16)	0.16869 (9)	0.0380 (4)	
O2	0.40602 (11)	0.62411 (17)	0.15533 (10)	0.0467 (5)	
O3	0.51971 (10)	0.55941 (14)	0.25061 (8)	0.0325 (4)	
O4	0.68947 (10)	0.49579 (18)	0.35584 (9)	0.0412 (4)	
O5	0.64533 (13)	0.66569 (18)	0.31577 (12)	0.0610 (6)	
C1	0.39872 (14)	0.5275 (2)	0.17983 (11)	0.0313 (5)	
C2	0.46290 (13)	0.4827 (2)	0.22686 (11)	0.0298 (5)	
C3	0.47776 (14)	0.3759 (2)	0.25122 (12)	0.0350 (5)	
H3	0.4478	0.3095	0.2425	0.042*	
C4	0.54854 (14)	0.3858 (2)	0.29291 (12)	0.0348 (5)	
H4	0.5740	0.3267	0.3169	0.042*	
C5	0.57159 (14)	0.4961 (2)	0.29108 (11)	0.0305 (5)	
C6	0.64101 (15)	0.5605 (2)	0.32343 (12)	0.0345 (5)	
O6	0.17981 (10)	0.52844 (16)	0.15262 (9)	0.0369 (4)	
O7	0.10622 (13)	0.36742 (18)	0.15767 (12)	0.0588 (6)	
O8	0.00890 (10)	0.46740 (14)	0.24548 (8)	0.0315 (4)	
O9	−0.14260 (11)	0.57111 (16)	0.34843 (10)	0.0405 (4)	
O10	−0.11593 (13)	0.39158 (18)	0.31678 (12)	0.0612 (6)	
C7	0.12146 (14)	0.4682 (2)	0.17212 (12)	0.0323 (5)	
C8	0.06639 (13)	0.5318 (2)	0.21566 (12)	0.0300 (5)	
C9	0.05879 (15)	0.6436 (2)	0.23144 (13)	0.0376 (6)	
H9	0.0908	0.7041	0.2176	0.045*	
C10	−0.00770 (15)	0.6518 (2)	0.27353 (13)	0.0377 (6)	
H10	−0.0277	0.7185	0.2924	0.045*	
C11	−0.03577 (13)	0.5440 (2)	0.28061 (11)	0.0297 (5)	
C12	−0.10354 (14)	0.4955 (2)	0.31801 (12)	0.0323 (5)	
N1	0.19302 (12)	0.4938 (2)	0.01174 (11)	0.0373 (5)	
N2	0.34234 (12)	0.4163 (2)	0.02842 (11)	0.0367 (5)	
C13	0.11996 (16)	0.5389 (3)	0.00524 (16)	0.0502 (7)	
H13	0.0945	0.5630	0.0430	0.060*	
C14	0.07967 (19)	0.5515 (3)	−0.05710 (18)	0.0649 (10)	
H14	0.0285	0.5841	−0.0602	0.078*	
C15	0.1155 (2)	0.5162 (4)	−0.11220 (18)	0.0689 (10)	
H15	0.0887	0.5234	−0.1534	0.083*	
C16	0.1937 (2)	0.4683 (3)	−0.10785 (15)	0.0551 (8)	
C17	0.2383 (3)	0.4298 (4)	−0.16290 (16)	0.0724 (11)	
H17	0.2149	0.4340	−0.2055	0.087*	
C18	0.3126 (3)	0.3879 (3)	−0.15429 (16)	0.0687 (10)	
H18	0.3399	0.3637	−0.1911	0.082*	
C19	0.35201 (19)	0.3792 (3)	−0.08942 (15)	0.0514 (7)	
C20	0.4306 (2)	0.3379 (3)	−0.07688 (19)	0.0665 (10)	
H20	0.4610	0.3124	−0.1116	0.080*	
C21	0.4615 (2)	0.3356 (3)	−0.0137 (2)	0.0688 (10)	
H21	0.5132	0.3073	−0.0050	0.083*	
C22	0.41626 (16)	0.3754 (3)	0.03824 (16)	0.0526 (8)	
H22	0.4388	0.3732	0.0812	0.063*	
C23	0.31052 (16)	0.4169 (2)	−0.03446 (13)	0.0385 (6)	
C24	0.23022 (17)	0.4602 (2)	−0.04374 (13)	0.0396 (6)	
N3	0.70653 (14)	0.5346 (2)	0.49399 (11)	0.0441 (6)	
N4	0.85693 (13)	0.6052 (2)	0.48709 (12)	0.0446 (5)	
C25	0.63292 (18)	0.4888 (4)	0.49529 (17)	0.0615 (9)	
H25	0.6080	0.4631	0.4559	0.074*	
C26	0.5921 (2)	0.4784 (4)	0.5545 (2)	0.0810 (13)	
H26	0.5412	0.4451	0.5542	0.097*	
C27	0.6266 (3)	0.5165 (4)	0.6111 (2)	0.0846 (14)	
H27	0.5987	0.5114	0.6500	0.101*	
C28	0.7044 (3)	0.5642 (3)	0.61325 (16)	0.0701 (11)	
C29	0.7473 (3)	0.6017 (4)	0.67048 (18)	0.0837 (14)	
H29	0.7229	0.5989	0.7111	0.100*	
C30	0.8223 (3)	0.6412 (3)	0.66776 (16)	0.0811 (14)	
H30	0.8484	0.6674	0.7066	0.097*	
C31	0.8645 (3)	0.6449 (3)	0.60622 (18)	0.0682 (10)	
C32	0.9437 (3)	0.6788 (4)	0.5992 (2)	0.0887 (14)	
H32	0.9738	0.7038	0.6364	0.106*	
C33	0.9790 (3)	0.6765 (4)	0.5388 (3)	0.0861 (14)	
H33	1.0323	0.6991	0.5351	0.103*	
C34	0.9324 (2)	0.6387 (3)	0.48203 (19)	0.0639 (9)	
H34	0.9554	0.6375	0.4408	0.077*	
C35	0.8235 (2)	0.6091 (3)	0.54787 (13)	0.0497 (7)	
C36	0.74293 (19)	0.5699 (3)	0.55125 (13)	0.0478 (7)	
O1W	0.23887 (10)	0.26884 (16)	0.10600 (9)	0.0363 (4)	
H1A	0.2766 (12)	0.245 (2)	0.1317 (12)	0.044*	
H1B	0.1959 (11)	0.275 (3)	0.1272 (12)	0.044*	
O2W	0.81807 (12)	0.34807 (19)	0.43587 (11)	0.0526 (5)	
H2A	0.8410 (17)	0.3280 (17)	0.3991 (11)	0.063*	
H2B	0.7755 (13)	0.3099 (16)	0.4451 (15)	0.063*	
O3W	0.30778 (18)	0.6991 (3)	0.05478 (13)	0.0923 (10)	
H3A	0.346 (2)	0.725 (2)	0.0796 (14)	0.111*	
H3B	0.324 (2)	0.696 (2)	0.0140 (10)	0.111*	
O41	0.6377 (2)	0.3137 (3)	0.07231 (14)	0.1025 (10)	
C41	0.6527 (3)	0.2954 (4)	0.1319 (2)	0.0811 (12)	
H41	0.6138	0.3170	0.1614	0.097*	
N41	0.71914 (18)	0.2478 (3)	0.15731 (15)	0.0652 (8)	
C42	0.7841 (3)	0.2158 (4)	0.1167 (2)	0.0884 (13)	
H42A	0.7954	0.1352	0.1224	0.106*	
H42B	0.8311	0.2599	0.1295	0.106*	
H42C	0.7693	0.2309	0.0710	0.106*	
C43	0.7313 (3)	0.2344 (5)	0.2284 (2)	0.1029 (17)	
H43A	0.7728	0.2862	0.2444	0.123*	
H43B	0.7471	0.1565	0.2384	0.123*	
H43C	0.6821	0.2517	0.2497	0.123*	

### Atomic displacement parameters (Å^2^)

**Table:**

	*U*^11^	*U*^22^	*U*^33^	*U*^12^	*U*^13^	*U*^23^
Cu1	0.02015 (14)	0.04554 (19)	0.02800 (15)	0.00042 (11)	0.00089 (11)	−0.00221 (13)
Cu2	0.02092 (15)	0.0551 (2)	0.02665 (15)	−0.00089 (12)	0.00027 (11)	−0.00304 (13)
O1	0.0264 (8)	0.0488 (11)	0.0381 (9)	−0.0065 (7)	−0.0071 (7)	0.0013 (8)
O2	0.0433 (10)	0.0450 (11)	0.0507 (11)	−0.0018 (9)	−0.0121 (9)	0.0099 (9)
O3	0.0292 (8)	0.0323 (9)	0.0350 (9)	−0.0030 (7)	−0.0098 (7)	0.0003 (7)
O4	0.0298 (9)	0.0540 (11)	0.0388 (10)	−0.0016 (8)	−0.0115 (8)	0.0034 (9)
O5	0.0560 (13)	0.0407 (12)	0.0833 (16)	−0.0055 (10)	−0.0351 (12)	−0.0044 (11)
C1	0.0266 (11)	0.0412 (14)	0.0259 (11)	0.0018 (10)	−0.0007 (9)	−0.0059 (10)
C2	0.0228 (10)	0.0384 (13)	0.0280 (11)	−0.0051 (9)	−0.0003 (9)	−0.0043 (10)
C3	0.0319 (12)	0.0349 (13)	0.0380 (13)	−0.0050 (10)	−0.0016 (10)	−0.0012 (11)
C4	0.0323 (12)	0.0369 (13)	0.0350 (12)	0.0014 (10)	−0.0027 (10)	0.0044 (11)
C5	0.0252 (11)	0.0370 (13)	0.0288 (11)	0.0002 (10)	−0.0050 (9)	−0.0004 (10)
C6	0.0302 (12)	0.0430 (15)	0.0296 (12)	−0.0007 (11)	−0.0058 (10)	−0.0038 (11)
O6	0.0271 (8)	0.0448 (11)	0.0395 (9)	−0.0021 (7)	0.0106 (7)	−0.0035 (8)
O7	0.0561 (13)	0.0419 (12)	0.0816 (16)	−0.0090 (10)	0.0394 (12)	−0.0160 (11)
O8	0.0282 (8)	0.0338 (9)	0.0334 (8)	0.0005 (7)	0.0106 (7)	−0.0026 (7)
O9	0.0346 (9)	0.0388 (10)	0.0494 (11)	0.0030 (8)	0.0188 (8)	−0.0034 (8)
O10	0.0621 (13)	0.0383 (11)	0.0867 (16)	−0.0095 (10)	0.0429 (13)	−0.0124 (11)
C7	0.0280 (11)	0.0409 (14)	0.0283 (11)	0.0035 (10)	0.0040 (9)	0.0012 (11)
C8	0.0229 (10)	0.0362 (13)	0.0313 (11)	−0.0007 (9)	0.0056 (9)	0.0054 (10)
C9	0.0326 (12)	0.0360 (14)	0.0448 (14)	−0.0007 (10)	0.0086 (11)	0.0062 (11)
C10	0.0361 (13)	0.0332 (13)	0.0448 (14)	0.0054 (10)	0.0130 (11)	−0.0004 (11)
C11	0.0249 (11)	0.0349 (13)	0.0295 (11)	0.0041 (9)	0.0043 (9)	−0.0009 (10)
C12	0.0268 (11)	0.0376 (14)	0.0328 (12)	0.0001 (10)	0.0045 (10)	−0.0025 (11)
N1	0.0281 (10)	0.0477 (13)	0.0360 (11)	−0.0030 (9)	−0.0001 (9)	0.0071 (10)
N2	0.0289 (10)	0.0427 (12)	0.0391 (11)	−0.0007 (9)	0.0068 (9)	−0.0002 (10)
C13	0.0308 (13)	0.065 (2)	0.0545 (17)	0.0023 (13)	−0.0026 (12)	0.0159 (15)
C14	0.0389 (16)	0.085 (3)	0.069 (2)	−0.0039 (16)	−0.0200 (16)	0.0269 (19)
C15	0.063 (2)	0.090 (3)	0.0510 (19)	−0.016 (2)	−0.0246 (17)	0.0140 (19)
C16	0.063 (2)	0.062 (2)	0.0390 (15)	−0.0121 (16)	−0.0092 (14)	0.0043 (14)
C17	0.102 (3)	0.084 (3)	0.0308 (15)	−0.016 (2)	−0.0045 (18)	−0.0045 (16)
C18	0.098 (3)	0.076 (3)	0.0338 (16)	−0.009 (2)	0.0204 (18)	−0.0112 (16)
C19	0.0619 (19)	0.0504 (18)	0.0436 (16)	−0.0086 (14)	0.0227 (14)	−0.0066 (13)
C20	0.064 (2)	0.070 (2)	0.069 (2)	−0.0006 (17)	0.0360 (19)	−0.0154 (18)
C21	0.0406 (17)	0.081 (3)	0.086 (3)	0.0116 (16)	0.0205 (17)	−0.012 (2)
C22	0.0337 (14)	0.067 (2)	0.0572 (18)	0.0096 (13)	0.0067 (13)	−0.0044 (16)
C23	0.0420 (14)	0.0387 (14)	0.0354 (13)	−0.0076 (11)	0.0082 (11)	−0.0020 (11)
C24	0.0449 (15)	0.0433 (15)	0.0304 (12)	−0.0112 (12)	−0.0012 (11)	0.0015 (11)
N3	0.0367 (12)	0.0621 (16)	0.0340 (11)	0.0132 (11)	0.0054 (9)	0.0039 (11)
N4	0.0375 (12)	0.0483 (14)	0.0468 (13)	0.0019 (10)	−0.0143 (10)	−0.0010 (11)
C25	0.0352 (15)	0.094 (3)	0.0561 (19)	0.0088 (16)	0.0133 (14)	0.0140 (18)
C26	0.052 (2)	0.128 (4)	0.065 (2)	0.020 (2)	0.0275 (19)	0.022 (2)
C27	0.074 (3)	0.121 (4)	0.062 (2)	0.042 (3)	0.038 (2)	0.019 (2)
C28	0.106 (3)	0.071 (2)	0.0335 (15)	0.039 (2)	0.0108 (18)	0.0031 (15)
C29	0.125 (4)	0.086 (3)	0.0391 (19)	0.035 (3)	0.002 (2)	−0.0050 (19)
C30	0.144 (4)	0.067 (2)	0.0304 (16)	0.027 (3)	−0.026 (2)	−0.0117 (15)
C31	0.095 (3)	0.0497 (19)	0.057 (2)	0.0103 (19)	−0.035 (2)	−0.0083 (16)
C32	0.123 (4)	0.070 (3)	0.069 (3)	−0.008 (3)	−0.049 (3)	−0.005 (2)
C33	0.065 (2)	0.071 (3)	0.118 (4)	−0.017 (2)	−0.044 (2)	0.003 (3)
C34	0.0469 (18)	0.068 (2)	0.075 (2)	−0.0107 (16)	−0.0198 (16)	0.0042 (18)
C35	0.073 (2)	0.0410 (15)	0.0330 (14)	0.0157 (15)	−0.0177 (14)	−0.0027 (12)
C36	0.0608 (19)	0.0508 (17)	0.0318 (13)	0.0207 (15)	0.0020 (13)	0.0019 (12)
O1W	0.0258 (8)	0.0385 (10)	0.0445 (10)	−0.0016 (8)	0.0001 (7)	0.0031 (8)
O2W	0.0401 (11)	0.0572 (13)	0.0609 (13)	−0.0075 (9)	0.0050 (10)	0.0044 (11)
O3W	0.087 (2)	0.128 (3)	0.0601 (16)	0.0616 (19)	−0.0142 (14)	−0.0162 (17)
O41	0.115 (2)	0.133 (3)	0.0584 (17)	0.014 (2)	−0.0119 (16)	0.0129 (18)
C41	0.077 (3)	0.102 (3)	0.065 (2)	0.016 (2)	0.005 (2)	−0.004 (2)
N41	0.0630 (17)	0.074 (2)	0.0591 (17)	0.0050 (15)	0.0052 (14)	−0.0023 (15)
C42	0.085 (3)	0.072 (3)	0.111 (3)	−0.008 (2)	0.033 (3)	−0.013 (3)
C43	0.090 (3)	0.149 (5)	0.069 (3)	0.038 (3)	−0.004 (2)	−0.002 (3)

### Geometric parameters (Å, º)

**Table:**

Cu1—O1	1.9475 (18)	C17—C18	1.332 (6)
Cu1—O6	1.9542 (17)	C17—H17	0.9300
Cu1—N1	2.022 (2)	C18—C19	1.442 (5)
Cu1—N2	2.030 (2)	C18—H18	0.9300
Cu1—O1W	2.3560 (19)	C19—C23	1.400 (4)
Cu1—O3W	2.911 (4)	C19—C20	1.404 (5)
Cu2—O9^i^	1.9449 (18)	C20—C21	1.353 (5)
Cu2—O4	1.9501 (18)	C20—H20	0.9300
Cu2—N3	2.029 (2)	C21—C22	1.394 (4)
Cu2—N4	2.031 (2)	C21—H21	0.9300
Cu2—O2W	2.415 (2)	C22—H22	0.9300
Cu2—O1W^ii^	2.639 (2)	C23—C24	1.431 (4)
O1—C1	1.270 (3)	N3—C25	1.335 (4)
O2—C1	1.232 (3)	N3—C36	1.345 (4)
O3—C2	1.369 (3)	N4—C34	1.321 (4)
O3—C5	1.375 (3)	N4—C35	1.368 (4)
O4—C6	1.263 (3)	C25—C26	1.403 (4)
O5—C6	1.233 (3)	C25—H25	0.9300
C1—O2	1.232 (3)	C26—C27	1.332 (6)
C1—C2	1.491 (3)	C26—H26	0.9300
C2—C3	1.352 (3)	C27—C28	1.405 (6)
C3—C4	1.422 (3)	C27—H27	0.9300
C3—H3	0.9300	C28—C29	1.400 (6)
C4—C5	1.337 (4)	C28—C36	1.430 (4)
C4—H4	0.9300	C29—C30	1.332 (6)
C5—C6	1.501 (3)	C29—H29	0.9300
C6—O4	1.263 (3)	C30—C31	1.453 (6)
O6—C7	1.271 (3)	C30—H30	0.9300
O7—C7	1.228 (3)	C31—C32	1.386 (6)
O8—C8	1.371 (3)	C31—C35	1.398 (4)
O8—C11	1.373 (3)	C32—C33	1.374 (6)
O9—C12	1.265 (3)	C32—H32	0.9300
O10—C12	1.223 (3)	C33—C34	1.424 (5)
C7—O7	1.228 (3)	C33—H33	0.9300
C7—C8	1.490 (3)	C34—H34	0.9300
C8—C9	1.343 (4)	C35—C36	1.419 (5)
C9—C10	1.425 (3)	O1W—H1A	0.843 (16)
C9—H9	0.9300	O1W—H1B	0.851 (16)
C10—C11	1.345 (4)	O2W—H2A	0.880 (16)
C10—H10	0.9300	O2W—H2B	0.862 (16)
C11—C12	1.491 (3)	O3W—H3A	0.846 (17)
N1—C13	1.324 (3)	O3W—H3B	0.878 (18)
N1—C24	1.358 (3)	O41—C41	1.236 (5)
N2—C22	1.324 (3)	C41—N41	1.319 (5)
N2—C23	1.353 (3)	C41—H41	0.9300
C13—C14	1.407 (4)	N41—C42	1.431 (5)
C13—H13	0.9300	N41—C43	1.449 (5)
C14—C15	1.347 (5)	C42—H42A	0.9600
C14—H14	0.9300	C42—H42B	0.9600
C15—C16	1.413 (5)	C42—H42C	0.9600
C15—H15	0.9300	C43—H43A	0.9600
C16—C24	1.408 (4)	C43—H43B	0.9600
C16—C17	1.432 (5)	C43—H43C	0.9600
			
O1—Cu1—O6	92.74 (8)	C16—C15—H15	119.8
O1—Cu1—N1	170.97 (8)	C24—C16—C15	116.3 (3)
O6—Cu1—N1	93.10 (8)	C24—C16—C17	118.3 (3)
O1—Cu1—N2	92.22 (8)	C15—C16—C17	125.4 (3)
O6—Cu1—N2	174.04 (9)	C18—C17—C16	121.3 (3)
N1—Cu1—N2	81.59 (9)	C18—C17—H17	119.3
O1—Cu1—O1W	88.27 (7)	C16—C17—H17	119.3
O6—Cu1—O1W	100.15 (7)	C17—C18—C19	121.8 (3)
N1—Cu1—O1W	97.48 (8)	C17—C18—H18	119.1
N2—Cu1—O1W	83.30 (8)	C19—C18—H18	119.1
O1—Cu1—O3W	95.85 (8)	C23—C19—C20	116.7 (3)
O6—Cu1—O3W	92.00 (7)	C23—C19—C18	118.5 (3)
N1—Cu1—O3W	77.06 (8)	C20—C19—C18	124.8 (3)
N2—Cu1—O3W	84.21 (8)	C21—C20—C19	119.3 (3)
O1W—Cu1—O3W	166.98 (7)	C21—C20—H20	120.3
O9^i^—Cu2—O4	97.76 (8)	C19—C20—H20	120.3
O9^i^—Cu2—O4	97.76 (8)	C20—C21—C22	120.3 (3)
O4—Cu2—O4	0.00 (13)	C20—C21—H21	119.8
O9^i^—Cu2—N3	171.30 (9)	C22—C21—H21	119.8
O4—Cu2—N3	90.43 (9)	N2—C22—C21	122.2 (3)
O4—Cu2—N3	90.43 (9)	N2—C22—H22	118.9
O9^i^—Cu2—N4	90.52 (9)	C21—C22—H22	118.9
O4—Cu2—N4	170.84 (9)	N2—C23—C19	123.7 (3)
O4—Cu2—N4	170.84 (9)	N2—C23—C24	116.7 (2)
N3—Cu2—N4	81.11 (10)	C19—C23—C24	119.6 (3)
O9^i^—Cu2—O2W	94.34 (7)	N1—C24—C16	123.0 (3)
O4—Cu2—O2W	91.11 (8)	N1—C24—C23	116.6 (2)
O4—Cu2—O2W	91.11 (8)	C16—C24—C23	120.5 (3)
N3—Cu2—O2W	88.39 (9)	C25—N3—C36	118.9 (3)
N4—Cu2—O2W	92.15 (9)	C25—N3—Cu2	127.6 (2)
O9^i^—Cu2—O1W^ii^	79.01 (7)	C36—N3—Cu2	113.2 (2)
O4—Cu2—O1W^ii^	96.69 (7)	C34—N4—C35	118.8 (3)
O4—Cu2—O1W^ii^	96.69 (7)	C34—N4—Cu2	129.0 (2)
N3—Cu2—O1W^ii^	97.21 (8)	C35—N4—Cu2	112.15 (19)
N4—Cu2—O1W^ii^	81.01 (8)	N3—C25—C26	121.8 (4)
O2W—Cu2—O1W^ii^	170.34 (6)	N3—C25—H25	119.1
C1—O1—Cu1	124.47 (16)	C26—C25—H25	119.1
C2—O3—C5	105.45 (18)	C27—C26—C25	119.7 (4)
C6—O4—Cu2	126.33 (18)	C27—C26—H26	120.2
O2—C1—O1	127.3 (2)	C25—C26—H26	120.2
O2—C1—O1	127.3 (2)	C26—C27—C28	121.3 (3)
O2—C1—C2	119.5 (2)	C26—C27—H27	119.4
O2—C1—C2	119.5 (2)	C28—C27—H27	119.4
O1—C1—C2	113.2 (2)	C29—C28—C27	125.7 (4)
C3—C2—O3	110.8 (2)	C29—C28—C36	118.4 (4)
C3—C2—C1	131.7 (2)	C27—C28—C36	115.9 (3)
O3—C2—C1	117.4 (2)	C30—C29—C28	121.2 (4)
C2—C3—C4	106.0 (2)	C30—C29—H29	119.4
C2—C3—H3	127.0	C28—C29—H29	119.4
C4—C3—H3	127.0	C29—C30—C31	122.2 (3)
C5—C4—C3	107.0 (2)	C29—C30—H30	118.9
C5—C4—H4	126.5	C31—C30—H30	118.9
C3—C4—H4	126.5	C32—C31—C35	115.4 (4)
C4—C5—O3	110.8 (2)	C32—C31—C30	126.2 (4)
C4—C5—C6	132.9 (2)	C35—C31—C30	118.3 (4)
O3—C5—C6	116.3 (2)	C33—C32—C31	121.8 (4)
O5—C6—O4	127.9 (2)	C33—C32—H32	119.1
O5—C6—O4	127.9 (2)	C31—C32—H32	119.1
O5—C6—C5	119.1 (2)	C32—C33—C34	118.7 (4)
O4—C6—C5	113.0 (2)	C32—C33—H33	120.6
O4—C6—C5	113.0 (2)	C34—C33—H33	120.6
C7—O6—Cu1	123.21 (16)	N4—C34—C33	120.9 (4)
C8—O8—C11	106.02 (18)	N4—C34—H34	119.5
C12—O9—Cu2^iii^	127.33 (17)	C33—C34—H34	119.5
O7—C7—O6	127.0 (2)	N4—C35—C31	124.2 (3)
O7—C7—O6	127.0 (2)	N4—C35—C36	116.9 (2)
O7—C7—C8	119.0 (2)	C31—C35—C36	118.9 (3)
O7—C7—C8	119.0 (2)	N3—C36—C35	116.6 (2)
O6—C7—C8	113.9 (2)	N3—C36—C28	122.3 (3)
C9—C8—O8	110.3 (2)	C35—C36—C28	121.0 (3)
C9—C8—C7	133.2 (2)	Cu1—O1W—H1A	105 (2)
O8—C8—C7	116.5 (2)	Cu1—O1W—H1B	96 (2)
C8—C9—C10	106.8 (2)	H1A—O1W—H1B	110 (2)
C8—C9—H9	126.6	Cu2—O2W—H2A	103.3 (14)
C10—C9—H9	126.6	Cu2—O2W—H2B	107.6 (14)
C11—C10—C9	106.4 (2)	H2A—O2W—H2B	116 (2)
C11—C10—H10	126.8	Cu1—O3W—H3A	111.3 (17)
C9—C10—H10	126.8	Cu1—O3W—H3B	108.4 (16)
C10—C11—O8	110.5 (2)	H3A—O3W—H3B	108 (2)
C10—C11—C12	132.6 (2)	O41—C41—N41	125.6 (4)
O8—C11—C12	117.0 (2)	O41—C41—H41	117.2
O10—C12—O9	127.1 (2)	N41—C41—H41	117.2
O10—C12—C11	119.4 (2)	C41—N41—C42	121.7 (4)
O9—C12—C11	113.5 (2)	C41—N41—C43	120.6 (3)
C13—N1—C24	118.6 (2)	C42—N41—C43	117.5 (4)
C13—N1—Cu1	128.8 (2)	N41—C42—H42A	109.5
C24—N1—Cu1	112.57 (17)	N41—C42—H42B	109.5
C22—N2—C23	117.8 (2)	H42A—C42—H42B	109.5
C22—N2—Cu1	129.7 (2)	N41—C42—H42C	109.5
C23—N2—Cu1	112.34 (17)	H42A—C42—H42C	109.5
N1—C13—C14	122.0 (3)	H42B—C42—H42C	109.5
N1—C13—H13	119.0	N41—C43—H43A	109.5
C14—C13—H13	119.0	N41—C43—H43B	109.5
C15—C14—C13	119.7 (3)	H43A—C43—H43B	109.5
C15—C14—H14	120.2	N41—C43—H43C	109.5
C13—C14—H14	120.2	H43A—C43—H43C	109.5
C14—C15—C16	120.4 (3)	H43B—C43—H43C	109.5
C14—C15—H15	119.8		
			
O6—Cu1—O1—C1	−101.97 (19)	C13—C14—C15—C16	−1.0 (6)
N2—Cu1—O1—C1	74.7 (2)	C14—C15—C16—C24	0.1 (5)
O1W—Cu1—O1—C1	157.95 (19)	C14—C15—C16—C17	−178.8 (4)
O3W—Cu1—O1—C1	−9.7 (2)	C24—C16—C17—C18	−0.4 (6)
O9^i^—Cu2—O4—O4	0.00 (13)	C15—C16—C17—C18	178.5 (4)
N3—Cu2—O4—O4	0.00 (12)	C16—C17—C18—C19	0.0 (6)
O2W—Cu2—O4—O4	0.00 (12)	C17—C18—C19—C23	−0.5 (5)
O1W^ii^—Cu2—O4—O4	0.00 (13)	C17—C18—C19—C20	−178.9 (4)
O9^i^—Cu2—O4—C6	−83.2 (2)	C23—C19—C20—C21	0.5 (5)
O4—Cu2—O4—C6	0 (17)	C18—C19—C20—C21	178.9 (4)
N3—Cu2—O4—C6	93.9 (2)	C19—C20—C21—C22	−0.9 (6)
O2W—Cu2—O4—C6	−177.7 (2)	C23—N2—C22—C21	1.0 (5)
O1W^ii^—Cu2—O4—C6	−3.4 (2)	Cu1—N2—C22—C21	175.2 (3)
O2—O2—C1—O1	0.0 (2)	C20—C21—C22—N2	0.2 (6)
O2—O2—C1—C2	0.0 (3)	C22—N2—C23—C19	−1.4 (4)
Cu1—O1—C1—O2	13.8 (4)	Cu1—N2—C23—C19	−176.6 (2)
Cu1—O1—C1—O2	13.8 (4)	C22—N2—C23—C24	179.3 (3)
Cu1—O1—C1—C2	−164.53 (15)	Cu1—N2—C23—C24	4.1 (3)
C5—O3—C2—C3	0.2 (3)	C20—C19—C23—N2	0.7 (4)
C5—O3—C2—C1	−178.82 (19)	C18—C19—C23—N2	−177.8 (3)
O2—C1—C2—C3	−166.5 (3)	C20—C19—C23—C24	179.9 (3)
O2—C1—C2—C3	−166.5 (3)	C18—C19—C23—C24	1.4 (4)
O1—C1—C2—C3	11.9 (4)	C13—N1—C24—C16	−1.8 (4)
O2—C1—C2—O3	12.3 (3)	Cu1—N1—C24—C16	179.2 (2)
O2—C1—C2—O3	12.3 (3)	C13—N1—C24—C23	177.2 (3)
O1—C1—C2—O3	−169.3 (2)	Cu1—N1—C24—C23	−1.8 (3)
O3—C2—C3—C4	−0.1 (3)	C15—C16—C24—N1	1.3 (4)
C1—C2—C3—C4	178.7 (2)	C17—C16—C24—N1	−179.7 (3)
C2—C3—C4—C5	0.0 (3)	C15—C16—C24—C23	−177.6 (3)
C3—C4—C5—O3	0.2 (3)	C17—C16—C24—C23	1.4 (4)
C3—C4—C5—C6	−179.4 (3)	N2—C23—C24—N1	−1.6 (4)
C2—O3—C5—C4	−0.2 (3)	C19—C23—C24—N1	179.1 (3)
C2—O3—C5—C6	179.4 (2)	N2—C23—C24—C16	177.4 (3)
O4—O4—C6—O5	0.0 (2)	C19—C23—C24—C16	−1.9 (4)
Cu2—O4—C6—O5	10.0 (4)	O4—Cu2—N3—C25	9.8 (3)
Cu2—O4—C6—O4	0 (100)	O4—Cu2—N3—C25	9.8 (3)
O4—O4—C6—C5	0.00 (13)	N4—Cu2—N3—C25	−173.8 (3)
Cu2—O4—C6—C5	−170.59 (15)	O2W—Cu2—N3—C25	−81.3 (3)
C4—C5—C6—O5	−176.0 (3)	O1W^ii^—Cu2—N3—C25	106.6 (3)
O3—C5—C6—O5	4.5 (4)	O4—Cu2—N3—C36	−176.3 (2)
C4—C5—C6—O4	4.5 (4)	O4—Cu2—N3—C36	−176.3 (2)
O3—C5—C6—O4	−175.0 (2)	N4—Cu2—N3—C36	0.2 (2)
C4—C5—C6—O4	4.5 (4)	O2W—Cu2—N3—C36	92.6 (2)
O3—C5—C6—O4	−175.0 (2)	O1W^ii^—Cu2—N3—C36	−79.5 (2)
O1—Cu1—O6—C7	−104.07 (19)	O9^i^—Cu2—N4—C34	−3.3 (3)
N1—Cu1—O6—C7	82.8 (2)	N3—Cu2—N4—C34	179.1 (3)
O1W—Cu1—O6—C7	−15.3 (2)	O2W—Cu2—N4—C34	91.1 (3)
O3W—Cu1—O6—C7	159.97 (19)	O1W^ii^—Cu2—N4—C34	−82.1 (3)
O7—O7—C7—O6	0.0 (3)	O9^i^—Cu2—N4—C35	176.79 (19)
O7—O7—C7—C8	0.0 (2)	N3—Cu2—N4—C35	−0.81 (19)
Cu1—O6—C7—O7	−2.0 (4)	O2W—Cu2—N4—C35	−88.8 (2)
Cu1—O6—C7—O7	−2.0 (4)	O1W^ii^—Cu2—N4—C35	98.01 (19)
Cu1—O6—C7—C8	179.69 (15)	C36—N3—C25—C26	1.1 (5)
C11—O8—C8—C9	0.3 (3)	Cu2—N3—C25—C26	174.8 (3)
C11—O8—C8—C7	−177.9 (2)	N3—C25—C26—C27	1.0 (6)
O7—C7—C8—C9	−167.6 (3)	C25—C26—C27—C28	−1.8 (7)
O7—C7—C8—C9	−167.6 (3)	C26—C27—C28—C29	−176.9 (4)
O6—C7—C8—C9	10.9 (4)	C26—C27—C28—C36	0.5 (6)
O7—C7—C8—O8	10.1 (4)	C27—C28—C29—C30	177.0 (4)
O7—C7—C8—O8	10.1 (4)	C36—C28—C29—C30	−0.3 (6)
O6—C7—C8—O8	−171.4 (2)	C28—C29—C30—C31	−1.8 (7)
O8—C8—C9—C10	−0.3 (3)	C29—C30—C31—C32	−176.4 (4)
C7—C8—C9—C10	177.5 (3)	C29—C30—C31—C35	2.2 (6)
C8—C9—C10—C11	0.2 (3)	C35—C31—C32—C33	−0.3 (6)
C9—C10—C11—O8	0.0 (3)	C30—C31—C32—C33	178.4 (4)
C9—C10—C11—C12	−180.0 (3)	C31—C32—C33—C34	0.4 (6)
C8—O8—C11—C10	−0.2 (3)	C35—N4—C34—C33	1.0 (5)
C8—O8—C11—C12	179.8 (2)	Cu2—N4—C34—C33	−178.9 (3)
Cu2^iii^—O9—C12—O10	12.0 (4)	C32—C33—C34—N4	−0.7 (6)
Cu2^iii^—O9—C12—C11	−167.91 (16)	C34—N4—C35—C31	−1.0 (5)
C10—C11—C12—O10	−179.1 (3)	Cu2—N4—C35—C31	178.9 (2)
O8—C11—C12—O10	0.9 (4)	C34—N4—C35—C36	−178.6 (3)
C10—C11—C12—O9	0.8 (4)	Cu2—N4—C35—C36	1.3 (3)
O8—C11—C12—O9	−179.1 (2)	C32—C31—C35—N4	0.7 (5)
O6—Cu1—N1—C13	1.5 (3)	C30—C31—C35—N4	−178.1 (3)
N2—Cu1—N1—C13	−175.8 (3)	C32—C31—C35—C36	178.2 (3)
O1W—Cu1—N1—C13	102.2 (3)	C30—C31—C35—C36	−0.6 (5)
O3W—Cu1—N1—C13	−89.8 (3)	C25—N3—C36—C35	175.0 (3)
O6—Cu1—N1—C24	−179.65 (18)	Cu2—N3—C36—C35	0.4 (3)
N2—Cu1—N1—C24	3.06 (18)	C25—N3—C36—C28	−2.4 (4)
O1W—Cu1—N1—C24	−78.98 (18)	Cu2—N3—C36—C28	−177.0 (2)
O3W—Cu1—N1—C24	89.01 (18)	N4—C35—C36—N3	−1.2 (4)
O1—Cu1—N2—C22	8.2 (3)	C31—C35—C36—N3	−178.9 (3)
N1—Cu1—N2—C22	−178.4 (3)	N4—C35—C36—C28	176.3 (3)
O1W—Cu1—N2—C22	−79.8 (3)	C31—C35—C36—C28	−1.5 (4)
O3W—Cu1—N2—C22	103.9 (3)	C29—C28—C36—N3	179.3 (3)
O1—Cu1—N2—C23	−177.29 (18)	C27—C28—C36—N3	1.6 (5)
N1—Cu1—N2—C23	−3.90 (18)	C29—C28—C36—C35	2.0 (5)
O1W—Cu1—N2—C23	94.72 (18)	C27—C28—C36—C35	−175.7 (3)
O3W—Cu1—N2—C23	−81.63 (18)	O41—O41—C41—N41	0.0 (3)
C24—N1—C13—C14	0.9 (4)	O41—C41—N41—C42	2.6 (7)
Cu1—N1—C13—C14	179.6 (2)	O41—C41—N41—C43	177.8 (5)
N1—C13—C14—C15	0.5 (5)		

Symmetry codes: (i) *x*+1, *y*, *z*; (ii) −*x*+1, *y*+1/2, −*z*+1/2; (iii) *x*−1, *y*, *z*.

### Hydrogen-bond geometry (Å, º)

**Table:**

*D*—H···*A*	*D*—H	H···*A*	*D*···*A*	*D*—H···*A*
O1*W*—H1*A*···O5^iv^	0.84 (2)	1.88 (2)	2.715 (3)	170 (3)
O1*W*—H1*B*···O7	0.85 (2)	1.96 (2)	2.729 (3)	151 (3)
O2*W*—H2*A*···O10^i^	0.88 (2)	1.98 (2)	2.729 (3)	143 (2)
O2*W*—H2*B*···O3*W*^iv^	0.86 (2)	1.89 (2)	2.726 (3)	163 (3)
O3*W*—H3*A*···O2	0.85 (2)	2.14 (3)	2.694 (3)	123 (2)
O3*W*—H3*B*···O41^v^	0.88 (2)	1.88 (2)	2.758 (4)	178 (4)
C3—H3···O5^iv^	0.93	2.53	3.424 (3)	161
C4—H4···O2^iv^	0.93	2.44	3.287 (3)	152
C9—H9···O10^vi^	0.93	2.33	3.193 (3)	155
C10—H10···O7^vi^	0.93	2.41	3.324 (3)	168
C15—H15···O8^vii^	0.93	2.42	3.327 (4)	166
C18—H18···O5^v^	0.93	2.56	3.416 (4)	154
C20—H20···O2^v^	0.93	2.52	3.230 (4)	133
C21—H21···O41	0.93	2.54	3.349 (5)	146
C33—H33···O2*W*^viii^	0.93	2.59	3.402 (5)	146

Symmetry codes: (i) *x*+1, *y*, *z*; (iv) −*x*+1, *y*−1/2, −*z*+1/2; (v) −*x*+1, −*y*+1, −*z*; (vi) −*x*, *y*+1/2, −*z*+1/2; (vii) −*x*, −*y*+1, −*z*; (viii) −*x*+2, −*y*+1, −*z*+1.
